# Erythrocyte Encapsulated Thymidine Phosphorylase for the Treatment of Patients with Mitochondrial Neurogastrointestinal Encephalomyopathy: Study Protocol for a Multi-Centre, Multiple Dose, Open Label Trial

**DOI:** 10.3390/jcm8081096

**Published:** 2019-07-24

**Authors:** Bridget E. Bax, Michelle Levene, Murray D. Bain, Lynette D. Fairbanks, Massimiliano Filosto, Sema Kalkan Uçar, Thomas Klopstock, Cornelia Kornblum, Hanna Mandel, Shamima Rahman, Agathe Roubertie, Mauro Scarpelli, Philip M. Sedgwick, Moshe Baru, Marcia Sellos-Moura, Jeanie Price, Patrick Horn, Niranjanan Nirmalananthan

**Affiliations:** 1 Molecular and Clinical Sciences, St. George’s, University of London, London SW17 0RE, UK; 2 The Purine Research Laboratory, St Thomas’ Hospital, London SE1 7EH, UK; 3 Centre for Neuromuscular Diseases, ASST Spedali Civili and University of Brescia, 25100 Brescia, Italy; 4 Division of Inborn Error of Metabolism, Ege University Medical Faculty, 35100 Izmir, Turkey; 5 Department of Neurology, Friedrich-Baur-Institute, University of Munich, Ziemssenstr. 1, 80336 Munich, Germany; 6 Munich Cluster for Systems Neurology (SyNergy), Ludwig Maximilians University, Geschwister-Scholl-Platz 1, 80539 Munich, Germany; 7 German Center for Neurodegenerative Diseases (DZNE), Feodor-Lynen-Strasse 17, 81377 Munich, Germany; 8 Department of Neurology, University Hospital Bonn, 53127 Bonn, Germany; 9 Galilee Medical Center, Nahariya 22100, Israel; 10 Mitochondrial Research Group, UCL London Great Ormond Street Institute of Child Health, London WC1N 1EH, UK; 11 Metabolic Unit, Great Ormond Street Hospital NHS Foundation Trust, London WC1N 3JH, UK; 12 Department of Pediatric Neurology, Centre Hospitalier Universitaire de Montpellier, 34295 Montpellier, France; 13 Institute of Neurology, University of Verona, 37126 Verona, Italy; 14 Institute for Medical and Biomedical Education, St George’s, University of London, London SW17 0RE, UK; 15 Orphan Technologies, Zürcherstrasse 19, 8640 Rapperswil, Switzerland; 16 Albireo Pharma, Inc., Boston, MA 02109, USA; 17 Department of Neurology, St George’s University Hospitals NHS Foundation Trust, London SW17 0QT, UK

**Keywords:** mitochondrial neurogastrointestinal encephalomyopathy, MNGIE, *TYMP*, enzyme replacement, erythrocyte encapsulated thymidine phosphorylase, thymidine phosphorylase, mitochondrial disease, rare disease, orphan disease, Phase II, multiple dose

## Abstract

Mitochondrial neurogastrointestinal encephalomyopathy (MNGIE) is an autosomal recessive disorder which primarily affects the gastrointestinal and nervous systems. This disease is caused by mutations in the nuclear *TYMP* gene, which encodes for thymidine phosphorylase, an enzyme required for the normal metabolism of deoxynucleosides, thymidine, and deoxyuridine. The subsequent elevated systemic concentrations of deoxynucleosides lead to increased intracellular concentrations of their corresponding triphosphates, and ultimately mitochondrial failure due to progressive accumulation of mitochondrial DNA (mtDNA) defects and mtDNA depletion. Currently, there are no treatments for MNGIE where effectiveness has been evidenced in clinical trials. This Phase 2, multi-centre, multiple dose, open label trial without a control will investigate the application of erythrocyte-encapsulated thymidine phosphorylase (EE-TP) as an enzyme replacement therapy for MNGIE. Three EE-TP dose levels are planned with patients receiving the dose level that achieves metabolic correction. The study duration is 31 months, comprising 28 days of screening, 90 days of run-in, 24 months of treatment and 90 days of post-dose follow-up. The primary objectives are to determine the safety, tolerability, pharmacodynamics, and efficacy of multiple doses of EE-TP. The secondary objectives are to assess EE-TP immunogenicity after multiple dose administrations and changes in clinical assessments, and the pharmacodynamics effect of EE-TP on clinical assessments.

## 1. Introduction

Mitochondrial neurogastrointestinal encephalomyopathy (MNGIE) is a fatal and rare autosomal recessive disorder of nucleotide metabolism caused by mutations in the nuclear thymidine phosphorylase gene (*TYMP*), which encodes cytosolic thymidine phosphorylase, the enzyme required for the normal metabolism of pyrimidine deoxynucleosides, thymidine, and deoxyuridine [1,2]. Pathogenic variants in *TYMP* result in a complete or partial absence of thymidine phosphorylase activity (<10% of healthy unaffected individuals), leading to an accumulation of thymidine and deoxyuridine in tissues and body fluids [3,4,5,6,7,8,9].

Elevated systemic concentrations of these deoxynucleosides lead to increased intracellular concentrations of their corresponding triphosphates. This perturbs the physiological equilibrium of the deoxynucleoside triphosphate pools within the mitochondria, thereby interfering with the normal replication of mitochondrial mtDNA, leading to multiple deletions, somatic point mutations and depletion of mtDNA [5,8,10,11], and ultimately mitochondrial failure [5,6,8]. It is believed that the consequent failure of cellular energy production directly causes the cardinal clinical manifestations through damage to the nervous and muscular systems.

Patients with MNGIE usually present during the second decade of life, although patients have presented as early as five months and as late as the fifth decade; the average age at diagnosis is 18.5 years [12]. The relatively late onset for a condition present at birth is thought to be due to the progressive accumulation of mtDNA defects, with the disease becoming apparent once the number of affected mitochondria reaches a critical threshold level. The disease is a multi-system disorder, and has a characteristic, although by no means universal, clinical presentation. Patients typically present with gastrointestinal symptoms including early satiety, nausea, dysphagia, gastroesophageal reflux, postprandial emesis, episodic abdominal pain, episodic abdominal distention, and diarrhoea. These symptoms are secondary to alimentary dysmotility caused by degeneration of the alimentary autonomic nervous system [2]. Patients generally have a thin body habitus with reduced muscle mass and cachexia. Episodes of frank intestinal pseudo obstruction may occur, and some patients develop a hepatopathy with liver steatosis and cirrhosis. Progressive external ophthalmoplegia and peripheral sensorimotor polyneuropathy are invariable. The latter affects the lower limbs initially and is typically demyelinating. On magnetic resonance imaging (MRI) there is, in the majority of cases, leukoencephalopathy with diffuse increased T2 signal in the deep white matter of the cerebral hemispheres, but this is generally believed to be asymptomatic [5,12].

MNGIE is a progressive disease, with patients dying at an average age of 37.5 years, and at present there are no approved therapies [4]. Allogeneic hematopoietic stem cell transplantation (HSCT) offers the possibility of a permanent correction of the thymidine phosphorylase deficiency. However, it is still highly experimental, carrying a mortality rate of approximately 63% [13]. Treatment with allogeneic HSCT is limited by the availability of a matched donor, and patients are often in poor clinical condition with impaired capacity to tolerate transplant related problems and the aggressive conditioning and immunosuppressive chemotherapy [13,14]. The administration of HSCT to patients with MNGIE presents pharmacological challenges in terms of administering drugs with possible mitochondrial toxicity, and the requirement for parenteral administration due to disturbed gastrointestinal function and impairment of absorption. A published consensus proposal for standardising an approach to allogeneic HSCT in patients with MNGIE recommends restricting the recruitment of patients with an optimal donor to those without irreversible end stage disease [13,14]. Patients who are oligosymptomatic are often reluctant to undergo HSCT due to its high mortality risk. Many patients are therefore ineligible for this treatment option and clinical management is based on symptom relief and palliation. A second experimental permanent treatment approach for MNGIE is orthotopic liver transplantation. Sustained normalisation of plasma thymidine and deoxyuridine concentrations have been reported in two patients who received liver transplantation [15]. A longitudinal evaluation of additional transplanted patients, however, is essential to confirm the clinical efficacy of this treatment approach. There is thus a critical requirement to develop an alternative treatment for these patients which would provide an expeditious normalization of nucleosides to prevent as much mitochondrial damage as possible.

**Figure 1:**
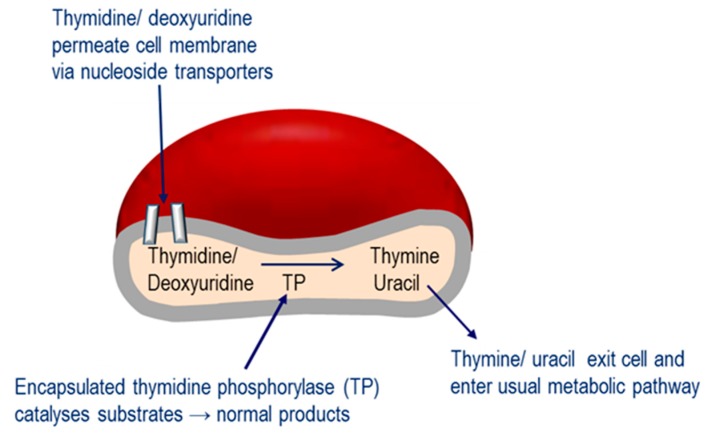
Mechanism of erythrocyte encapsulated thymidine phosphorylase (EE-TP) action. Plasma thymidine and deoxyuridine diffuse across the erythrocyte membrane via nucleoside transporters into the erythrocyte cytosol where the encapsulated thymidine phosphorylase (TP) catalyses their metabolism to thymine and uracil, which then exit the erythrocyte to enter the normal metabolic pathways.

The Investigational Medicinal Product under investigation in this study is erythrocyte encapsulated thymidine phosphorylase (EE-TP), which is produced under Good Manufacturing Practice by the ex vivo encapsulation of recombinant *Escherichia coli* thymidine phosphorylase into patient’s autologous erythrocytes using an automated red cell loader device [16]. EE-TP is intravenously infused into the patient where it aims to correct the fundamental lesion in MNGIE by replacement of the deficient thymidine phosphorylase. The rationale for the development of EE-TP is based on thymidine and deoxyuridine being able to diffuse across the erythrocyte membrane via nucleoside transporters into the cytosol where the encapsulated enzyme catalyses their metabolism to the normal products, thymine and uracil, respectively (Figure 1). The products then exit the cell into the blood plasma where they are further metabolised as normal. It is proposed that regular intravenous (IV) administrations of EE-TP to patients with MNGIE will lead to a sustained reduction or elimination of plasma thymidine and deoxyuridine, leading to a clearance from the cellular compartments and thus an amelioration of the intracellular deoxynucleotide imbalances. This should prevent further damage to mtDNA. By relieving the nervous system and muscle of the toxic effects of the accumulated metabolites, EE-TP aims to arrest and reverse the progression of the clinical disease.

EE-TP has the advantage of prolonging the circulatory half-life of the enzyme to that of the erythrocyte half-life (19–29 days) and minimising immunogenic reactions, which are often observed in enzyme replacement therapies administered by the conventional route.

Clinical experience with EE-TP is limited to a proof-of-concept study in a single patient diagnosed with MNGIE and a compassionate clinical evaluation in four patients with MNGIE [17,18,19]. EE-TP was well tolerated and reductions in the disease-associated plasma metabolites, thymidine and deoxyuridine, were observed in all patients. Clinical improvements were observed in three patients who received long-term treatment, suggesting that EE-TP is able to reverse some aspects of the disease pathology. Transient, non-serious adverse events were observed in two of the five patients; these did not lead to therapy discontinuation and were managed with pre-medication prior to infusion of EE-TP. Despite using an *E. coli* recombinant protein, specific anti-thymidine phosphorylase antibodies were detected in only one patient.

The aim of this study is to investigate the safety, tolerability, pharmacodynamics, and efficacy of EE-TP in patients with MNGIE. We hypothesise that treatment with EE-TP will arrest and reverse the progression of the clinical disease. The study protocol reported here (version 6.0) was written in compliance with the Standard Protocol Items: Recommendations for Interventional Trials (SPIRIT) 2013 [20]. The sponsor, St George’s, University of London, requested scientific assistance for EE-TP and the current protocol was updated in line with the European Medicines Agency (EMA) advice provided. The study will be conducted in accordance with the following and all subsequent amendments: (i) International Council for Harmonisation of Technical Requirements for Registration of Pharmaceuticals for Human Use (ICH) E6: Good Clinical Practice: Consolidated guideline Committee for Proprietary Medicinal Products (CPMP)/ICH/135/95 (July 1996), adopted in the EU by CPMP, (ii) European Commission Directive 2001/20/EC (April 2001), (iii) European Commission Directive 2003/94/EC (October 2003), (iv) European Commission Directive 2005/28/EC (April 2005), (v) Manufacture of Investigational Medicinal Products: Volume 4, Annex 13 of the EU Guidelines to Good Manufacturing Practice (February 2010), and (vi) The Medicines for Human Use (Clinical Trials) Regulations 2004 and all subsequent amendments.

## 2. Methods

### 2.1. Trial Objectives

The primary objectives of this study are to determine the safety, tolerability, pharmacodynamics, and efficacy (as measured by weight stabilisation) of multiple doses of EE-TP in patients with MNGIE. The secondary objectives are to assess the immunogenicity of EE-TP after multiple dose administrations, evaluate changes in clinical assessments, and assess the pharmacodynamics effect of EE-TP on clinical assessments. Additional exploratory objectives aim to evaluate the effects of EE-TP on serum and plasma markers of mitochondrial condition, MRI (brain), abdominal ultrasound, electromyography, and neuro-ophthalmological assessments.

### 2.2. Study Drug

EE-TP will be prepared by trained personnel using an automated red cell loading process at each trial site according to the Red Cell Loader protocol. A predetermined volume of the patient’s blood (120 to 360 mL) will be removed from the patient and then introduced into the Red Cell Loader, which separates the blood components and then subjects the erythrocytes to a reversible hypo-osmotic dialysis process. Under hypo osmotic conditions, the erythrocytes swell due to an influx of water and at a critical size, pores form in the membrane. Whilst in a permeable state, the study drug, thymidine phosphorylase, enters the erythrocytes by diffusion. The permeability was reversed by restoration of iso-osmotic conditions, encapsulating the thymidine phosphorylase within the erythrocytes, to form EE-TP. The EE-TP will be dispensed into a sterile European Pharmacopoeia grade 100 mL capacity blood transfer bag and released as multiple units (2–6 bags, the number depending on the dose to be administered) for infusion within 30 min of manufacture. Characteristics of EE-TP are shown in Table 1.

### 2.3. Trial Design

**Figure 2:**
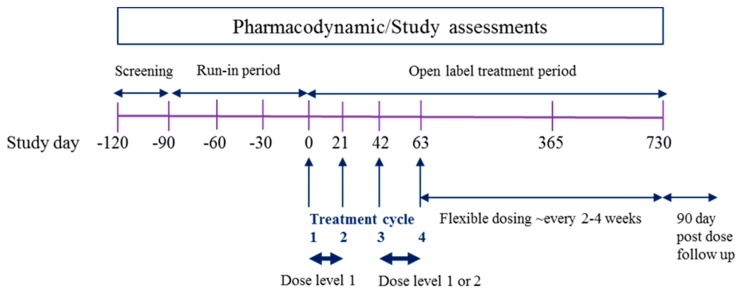
Study Design Schematic. The overall study duration will be 31 months, comprising 28 days screening, 90 days run-in, 24 months open label treatment and 90 days post-dose follow-up. The first 4 treatment cycles will be administered every 21 days, with patients receiving Dose Level 1 for the first 2 treatment cycles. If metabolic correction is not achieved, the subsequent 2 treatment cycles will be administered at Dose Level 2. From treatment day 78, a flexible dosing approach will be employed to achieve metabolic correction, where administration will be at Dose Levels 1, 2 or 3, once every 2–4 weeks ± 2 days until the end of the study.

The trial is a Phase 2, multi-centre, multiple dose, open label trial without a control which will be conducted in a maximum of five sites in Europe and Israel, with all patients receiving EE-TP. For each patient, the overall study duration will be 31 months, consisting of a 28 day screening phase, a 90 day run-in phase, a 24 month open-label treatment phase, and a 90 day follow-up phase. A schematic of the proposed study design is presented in Figure 2.

Patients will be screened for eligibility from Day −120 to Day −92. If eligible, patients will then enter the run-in period, from Day −91 to pre-dose on Day 0, in which key aspects of their overall clinical condition and metabolite levels will be recorded. The variability in these clinical and metabolic parameters is currently unknown, and these data will primarily serve to act as each patient’s own pre-treatment/control observations.

Prior to entering the treatment phase, patients will attend the study site for pre-treatment assessments and to determine continued study eligibility either the day before dosing (Day –1) or in the morning of the day of dosing (Day 0). For the first four treatment cycles, the administration will be every 21 days, with the administration of EE-TP at the same time of day (±2 h). The first dose (Treatment Cycle 1) of EE-TP will be administered on day 0, with subsequent doses on Days 21, 42, and 63 (Treatment Cycles 2, 3 and 4, respectively). For Treatment Cycles 1, 2, 3, and 4, patients will leave the study site following dosing and return the next day (Days 1, 22, 43, and Day 64, respectively) for 24 h post-dose assessments. The planned dose levels are presented in Table 2. All patients will receive infusions at Dose Level 1 (~58–65 × 10^10^ erythrocytes encapsulating ~30–49 U TP/10^10^ erythrocytes) for the first 2 treatment cycles (Treatment Cycles 1 and 2). If metabolic correction (defined as plasma thymidine <3 µmol/L and deoxyuridine <5 µmol/L, i.e., below the diagnostic levels for MNGIE) is not achieved, the subsequent 2 treatment cycles (Treatment Cycles 3 and 4) will be administered at Dose Level 2 (~58–65 × 10^10^ erythrocytes encapsulating ~50–69 U TP/10^10^ erythrocytes).

From Day 78, the dose will remain at the dose that achieved metabolic correction during the initial treatment phase (pre-Day 78). If metabolic correction was not achieved, treatment will advance to Dose Level 3 (~58–65 × 10^10^ erythrocytes encapsulating ~70–90 U TP/10^10^ erythrocytes) for subsequent treatment cycles. It is planned that patients will receive EE-TP every 2–4 weeks ± 2 days until the end of the study. Dose frequency may be reduced (e.g., from every 2 weeks to every 3–4 weeks) for individual patients based on emerging safety, tolerability, efficacy, and pharmacodynamics data. The interval between doses will not be shorter than 2 weeks ± 2 days. In the advent of an increase in plasma metabolite levels, the frequency of dosing and/or dose level will be reviewed and adjusted accordingly.

**Figure 3:**
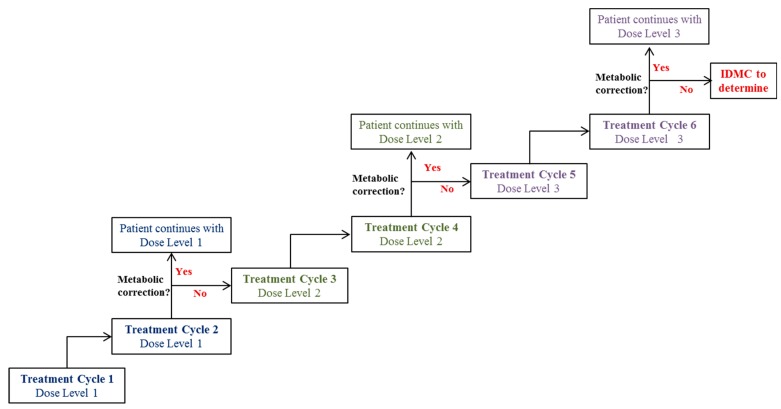
Planned dosing scheme during EE-TP treatment phase. All patients will receive infusions at Dose Level 1 for the first 2 treatment cycles (Treatment Cycles 1 and 2). If metabolic correction is not achieved, the subsequent 2 treatment cycles (Treatment Cycles 3 and 4) will be administered at Dose Level 2. If metabolic correction is not achieved, treatment will advance to Dose Level 3 in Treatment Cycles 5 and 6. If metabolic correction is not achieved after Treatment Cycle 6 at Dose Level 3, the Independent Data Monitoring Committee (IDMC) will provide recommendations on the need for dosing modifications.

The study will be flexible; continued pharmacodynamic assessment will enable dose optimisation and further inform dose response models and establish the therapeutic window for the treatment. A Patient Oversight Committee will perform ongoing reviews of safety, tolerability, efficacy, and available pharmacodynamic data to ensure the acceptability of continued dosing and/or dose progression for each patient. An overview of the dosing scheme is presented in Figure 3.

### 2.4. Participants

The study will enrol 12 adult male or female patients with MNGIE, aged 18 years or above, of any race and who have received no previous treatments. An additional 8 juvenile patients will be enrolled following an Independent Data Monitoring Committee review of an interim analysis of safety data safety and tolerability data. Up to four patients aged 16 years or older may be dosed at the lowest level after 24 patient-months of exposure in patients aged 18 years or over. The starting dose will be Dose Level 1 for both groups, with escalation to the next dose level as described for the adult cohort. Up to four patients aged 12 years or older may be dosed at the lowest dose level (Dose Level 1) after 24 patient-months of exposure in patients aged under 18 years. Juvenile cohorts may escalate to a higher dose or de-escalate to a lower and/or intermediate dose level(s) of EE-TP previously administered. Screening failures and patients withdrawing from the study may be substituted with Independent Data Monitoring Committee recommendations.

#### 2.4.1. Study Inclusion Criteria

1. Patients must be aged 18 years or older. The age range will be extended to include:
  (i) Patients aged 16 years or older after 24 patient-months of exposure in those aged 18 years or over;
  (ii) Patients aged 12 years or older after 24 patient-months of exposure in patients aged under 18 years of age at the time of enrolment.
2. Patients must be diagnosed with MNGIE by demonstrating all of the following:
  (i) <18% normal thymidine phosphorylase activity in the buffy coat;
  (ii) >3 μmol/L plasma thymidine;
  (iii) >5 μmol/L plasma deoxyuridine;
  (iv) Confirmation of the presence of biallelic pathogenic variants in TYMP by sequencing.
3. Patients must be able to undergo study procedures.
4. Patients must agree to either remain completely true abstinent or to use 2 effective contraceptive methods from Screening until completion of the Follow-up Visit.
5. Patients must be willing to sign and date the written Informed Consent Form.

#### 2.4.2. Study Exclusion Criteria

6. Patients who have received a successful liver or bone marrow transplant.
7. Patients suitable for allogeneic HSCT.
8. Patients with a matched allogeneic HSCT donor.
9. Patients with a history of human immunodeficiency virus, hepatitis B infection, or an active hepatitis C infection.
10. Patients who are severely disabled or with a life expectancy of less than 12 months at Screening based on the opinion of the investigator.
11. Female patients who are:
  (i) Pregnant, planning a pregnancy, or are unwilling to use contraception;
  (ii) Breastfeeding or lactating.
12. Patients who have donated blood in the 90 days prior to Screening.
13. Patients with a confirmed red blood cell count of <3.0 × 10^9^ per mL.
14. Patients who have a significant history of alcoholism or drug/chemical abuse within 1 year prior to Screening based on the opinion of the investigator.
15. Patients who have an abnormality in heart rate, blood pressure, or body temperature at Screening that increases the risk of participating in the study based on the opinion of the investigator.
16. Patients who have an abnormality in the 12-lead electrocardiogram at Screening that increases the risk of participating in the study based on the opinion of the investigator.
17. Patients who have, or have a history of, any clinically significant neurological, gastrointestinal, renal, hepatic, cardiovascular, psychiatric, respiratory, metabolic, endocrine, haematological, or other major disorder (except for disorders associated with MNGIE that do not constitute a risk when taking the study drug and would not interfere with the study objectives) based on the opinion of the investigator.
18. Patients with any current malignancy, or a history of malignancy within 5 years prior to Screening, with the exception of adequately treated or excised non-metastatic basal cell or squamous cell cancer of the skin or cervical carcinoma in situ.
19. Patients who are currently enrolled in, are planning to participate in, or have discontinued within the last 30 days from a clinical study involving an investigational medicinal product or are concurrently enrolled in medical research judged not to be scientifically or medically compatible with EE-TP.
20. Patients with any medical condition which would make the patient unsuitable for enrolment or could interfere with the patient’s participation in, or completion of, the study based on the opinion of the investigator.

### 2.5. Interventions

#### 2.5.1. Preparation and Administration of Erythrocyte Encapsulated Thymidine Phosphorylase (EE-TP)

On the morning of each treatment cycle patients will undergo venesection for the collection of 120–360 mL of blood for the manufacture of EE-TP. The volume of blood collected will be dependent on the patient’s haematocrit, red cell count, and the dose of EE-TP to be administered. EE-TP will be administered as an intravenous infusion, within 30 min of the completion of manufacture, according to the specified study protocol, using a standard intravenous infusion set with a micro-aggregate filter in-line. 

#### 2.5.2. Assessments

The following information will be recorded for all potential patients as part of the screening assessments: Informed consent, inclusion/exclusion criteria, demography, including sex, race and age, medical history, and concomitant medication use (Table 3).

Each patient will be required to undergo the following assessments during the screening and run-in phases (Table 3) and treatment and follow-up phases (Table 4) at the study times indicated: Vital signs measurement, 12-lead electrocardiogram (ECG), body weight (kg) and height (m), physical examination, pregnancy test (female patients only), adverse event recording, total parenteral nutrition (TPN) use, handgrip strength using handgrip dynamometry, disability measured using the Rasch built Overall Disability Scale (RODS), ambulatory function measured using the 10 m walk test, gastrointestinal symptoms (abdominal pain, diarrhoea, disrupted swallowing, gas and bloating, gastroesophageal reflux, and nausea and vomiting) measured using the Patient-Reported Outcomes Measurement Information System (PROMIS) short form scales, distal sensory impairment and areflexia or motor strength using neurological examinations, MRI brain, abdominal ultrasound, nerve conduction studies and electromyography, neuro-ophthalmology and quality of life measured using EuroQol 5 Dimension (EuroQol-5D), Clinical Global Impression-Improvement Scale (CGI-I), Patient Global Impression of Change (PGIC), and visual analogue scale (VAS) for determining the severity of the patient’s most disabling symptom.

#### 2.5.3. Clinical Laboratory and Pharmacodynamic Evaluations

Blood and urine samples will be collected for haematology, serum biochemistry and urinalysis evaluations and for the longitudinal monitoring of thymidine and deoxyuridine concentrations at the times indicated in Table 3 and Table 4. Blood samples will also be collected for the measurement of anti-thymidine phosphorylase antibodies and neutralizing antibodies, and for the exploratory analysis of fibroblast growth factor 21 (FGF21), growth differentiation factor 15 (GDF15), mtDNA, messenger RNA (mRNA), microRNA (miRNA), and lipidomics, proteomics, and other markers of mitochondrial condition (Table 3 and Table 4).

### 2.6. Trial Endpoints

#### 2.6.1. Efficacy Endpoints

The primary efficacy endpoint will be the mean absolute change from baseline in body mass index (BMI) at 24 months. The secondary efficacy endpoints will be the mean absolute change from baseline in BMI at Day 63 and 3, 6, 9, 12, 15, 18, and 21 months, plus the change from baseline at 63 days and 3, 6, 9, 12, 15, 18, 21, and 24 months in: (i) The proportion of patients who require TPN; (ii) Handgrip dynamometry; (iii) RODS; (iv) 10 m walk test; (v) EuroQo-5D; (vi) CGI-I; (vii) PROMIS short form scales; (viii) PGIC; (ix) Neurological examination tests; and (x) VAS for the most disabling symptom.

#### 2.6.2. Safety Endpoints

The clinical safety of EE-TP will be evaluated by: (i) The incidence, frequency, and severity of adverse events (or treatment-emergent adverse events); (ii) The incidence of laboratory abnormalities, based on haematology, serum biochemistry, and urinalysis test results; (iii) Vital sign measurements (systolic and diastolic blood pressure, pulse rate, respiratory rate, and body temperature); (iv) 12 lead ECG parameters; (v) Physical examination findings; and (vi) The use of concomitant medication(s).

### 2.7. Sample Size

The sample size for this study is dictated by the rarity of the condition and not by any quantitative statistical considerations or formal sample size calculations. At least 12 adult treatment naïve patients will be treated in this study, with the option to enrol an additional 8 juvenile patients, beyond this original set of 12 patients.

### 2.8. Statistical Analyses

Statistical analyses will be primarily descriptive. Analyses will be performed using Statistical Analysis Software^®^ (Version 9.2 or higher, SAS Institute, Cary, NC, USA). For continuous data, summary statistics will include the arithmetic mean, arithmetic standard deviation, median, lower and upper quartiles, plus minimum, maximum, and number. For log normal data, summary statistics, including the geometric mean and geometric coefficient of variation, will also be presented. For categorical data, frequency counts and percentages will be presented. Data listings will be provided for all patients up to the point of withdrawal, with any patients excluded from the relevant population indicated. For the calculation of summary statistics and statistical analysis, unrounded data will be used.

For each study participant the change from baseline will be calculated by subtracting their mean baseline value from the value at the specific time point. The mean change from baseline will be derived as the arithmetic mean of all individual patients’ change from baseline values.

#### 2.8.1. Safety Data Analysis

A baseline sign and symptom will be defined as an adverse event that starts after the patient has provided written informed consent and resolves prior to the first treatment cycle. It will also be defined as an adverse event that starts prior to the first treatment cycle and does not increase in severity after dosing. A treatment emergent adverse event will be defined as an adverse event that occurs post-dose or that is present pre-dose and becomes more severe post-dose. Treatment emergent adverse events will be summarised by dose level, maximum severity, and relationship to the study drug. The frequency of treatment emergent adverse events plus the number and percentage of patients experiencing a treatment emergent adverse event will be summarised by dose level, the Medical Dictionary for Regulatory Activities system organ class, plus preferred term. A frequency summary will be presented by day of onset across the multiple dosing period. 

Vital signs, serum biochemistry, haematology, and urinalysis data will be summarised by dose level, together with changes from the baseline. All serum biochemistry, haematology, and urinalysis data outside the clinical reference ranges will be listed by parameter and treatment. ECG data, including QTc, QTcB, QTcF, the PR and QT intervals, QRS duration, and heart rate, will be obtained directly from the 12-lead ECG traces. The ECG data will be summarised by dose level. Values for ECG parameters outside the clinical reference ranges will be indicated on the individual patient data listings. Physical examination and concomitant medication data will be listed.

#### 2.8.2. Pharmacodynamic Data Analysis

The absolute values and changes from baseline urine and plasma concentrations of thymidine and deoxyuridine will be calculated using Day 0 or Day 1 as the baseline. The proportion of patients who achieve metabolic correction (plasma thymidine <3 µmol/L and deoxyuridine <5 µmol/L) will be presented by dose. The activity of thymidine phosphorylase administered will be calculated for each treatment cycle and longitudinal changes will be plotted. The presence of specific anti-thymidine phosphorylase antibodies will be recorded.

#### 2.8.3. Efficacy Data Analysis

The changes from baseline for all efficacy endpoints and outcomes will be calculated using data from the run-in period (Day −91 to Day 0) as the baseline. Absolute and percentage changes from baseline in BMI will be plotted. In addition, absolute and percentage changes from baseline in BMI will be recorded against plasma and urine concentrations of thymidine and deoxyuridine. The percentage of patients achieving at least the minimum threshold of weight gain will be presented by dose. Since juvenile patients in the juvenile cohorts will be developing (i.e., growing and maturing) during the study, changes in weight and other efficacy endpoints cannot be reliably attributed to EE-TP, and therefore their data will not be included in the main efficacy analyses, and will be analysed separately.

#### 2.8.4. Exploratory Data Analysis

Overall change from baseline to 12 and 24 months, categorised as improved, stable, and deteriorated, will be carried out at the following clinical assessments: (i) MRI (brain); (ii) Abdominal ultrasound; (iii) Nerve conduction studies and electromyography; and (iv) Neuro ophthalmological assessment. In addition, changes from baseline in serum or plasma biomarkers related to mitochondrial health such as FGF 21, GDF15, and other markers of mitochondrial condition at Days 0, 1, 7, 14, 21, 28, 35, 42, 43, 49, 56, 63, 64, 70, and 77, and at each clinic visit from Day 78 until the end of the study, will be recorded. Associations between pharmacodynamics parameters and clinical activity parameters will be explored.

#### 2.8.5. Interim Analysis

An interim review of data will be conducted after each of the twelve-adult treatment naïve patients has been exposed to EE-TP for at least one year. This interim time point has been chosen to assess and confirm the potential utility of EE-TP in this population, and to provide potential early access of EE-TP in this population. The exposure for a patient at the interim stage could exceed one year if a patient is recruited early on into the study.

### 2.9. Data Monitoring Committee

The appointed Independent Data Monitoring Committee will oversee safety throughout the trial period. The committee will comprise a chairperson, a specialist in MNGIE, and a statistician. The purpose of the committee will be to review unblinded study information including protocol violations, patient withdrawals, adverse/serious adverse events, and laboratory data. An assessment will be made by the Independent Data Monitoring Committee with regard to the enrolment of juvenile patients.

### 2.10. Patient Confidentiality

Patient confidentiality will be held by the participating investigators and all personnel involved at the study sites. Following Good Clinical Practice principles, a patient number will be used to identify the patient in the study records. Laboratory samples and any samples in storage will be labelled using only the patient number, and the identity of the patient will be known only by the study site. The numbering code associated with the labels will be held by the study sites, thereby allowing no unwarranted access to the information. When reporting results for any interim safety information, analyses, and end of the study, the code will be shared as per sponsor standard operating procedures. The numbering code will also be held for any samples in storage until marketing approval in the countries where the study is conducted, or until notification is received that storage is no longer required.

### 2.11. Dissemination

The results of this study will be disseminated through presentation at international scientific conferences and reported in peer-reviewed scientific journals. We also intend to disseminate our results to the rare disease community.

### 2.12. Ethical Conduct and Approval and Trial Registration

This study will be conducted in accordance with consensus ethics principles derived from the Declaration of Helsinki. Approval for the operation of the clinical trial at the United Kingdom study site was granted following Research Ethics Committee review (18/SW/0266) and Health Research Authority (HRA) and Health and Care Research Wales (HCRW) review. For the non-United Kingdom trial sites, the study will be considered by the relevant Ethics Committee (EC) of each participating country site.

### 2.13. Trial Registration and Status

The trial is registered at ClinicalTrials.gov, with identifier number NCT03866954. The trial is in preparation and is not yet open for participant recruitment.

## 3. Discussion

Mitochondrial neurogastrointestinal encephalomyopathy is a relentlessly progressive disorder with an invariably fatal outcome, and therefore an early diagnosis and subsequent normalisation of deoxynucleosides is critical to avoid ongoing mitochondrial damage and disease progression. The compassionate treatment of patients with regular infusions of EE-TP demonstrated a reduction or elimination of plasma deoxynucleosides and clinical improvements in patients who received long-term treatment, suggesting that EE-TP is able to reverse some aspects of the disease pathology [18,19]. This study aims to determine the safety, tolerability, pharmacodynamics, and efficacy of multiple doses of EE-TP in patients with MNGIE. In practice, treatment with EE-TP is likely to be sought for patients in whom the risk of mortality from allogeneic HSCT would be too high, and also for those for whom there is no matched donor. Treatment with EE-TP may also be indicated as a rescue or maintenance therapy prior to the availability of a suitable HSCT donor, and for patients who are oligosymptomatic and reluctant to undergo HSCT.

The total sample size of 12 adult treatment naïve patients is not based on a formal statistical calculation; it is a relatively small sample size, but this is expected to be offset by the nature of the condition. MNGIE has a predictable clinical course; it is relentlessly progressive, with a very poor prognosis for life, and it has been reported that the mean age of death is 37.6 years (range 15–54 years) [21]. Therefore, any arrest or improvement of patients’ clinical deficits and/or reduction in mortality should be conspicuous and would not be plausibly explained by factors other than treatment with EE-TP. Clearly, such findings would be highly clinically relevant.

The rationale for the dose selection is based on data generated from patients who received EE-TP on a compassionate treatment basis. This indicated that the optimal parameters required to achieve metabolic depletion of thymidine and deoxyuridine were a maximum of 90 U TP/10^10^ erythrocytes and a minimum 58 × 10^10^ erythrocytes. We therefore propose the infusion of an optimum number of ~58–65 × 10^10^ erythrocytes at three dose levels: 30–49, 50–69 and 70–90 U/10^10^ erythrocytes. The starting dose for all patients will be Dose Level 1; this will be administered for at least two treatment cycles (three weeks apart). This initial three week period has been designed to ensure metabolic correction is captured appropriately, and dosing changes can be made accordingly. If metabolic correction is observed, the patient will continue to receive this same low dose. However, if no metabolic correction is observed then the patient will receive Dose Level 2 (mid dose level) for two consecutive treatment cycles. If metabolic correction is still not achieved, treatment will advance to Dose Level 3 (high dose) for subsequent treatment cycles. Both dose and dosing regimens will be flexible.

The study will be open label with a three month run-in period, which is appropriate, as the primary objective is to evaluate the safety, tolerability, efficacy, and pharmacodynamics of EE-TP in patients with MNGIE. Currently, it is not known how variable or what change, if any, in the clinical and metabolic parameters will be observed over this three month run-in period, but these data will primarily serve to act as each patient’s own pre-treatment/control observations. Patients with MNGIE present with a wide variety of clinical (gastrointestinal and neurological) features and these data are highly likely be very heterogeneous between patients. Nonetheless, prolonged treatment with EE-TP is expected to cause positive changes in these various clinical and metabolic endpoints. The decision not to include a placebo control group was taken due to the serious and life-threatening nature of the disease and the evidence available from compassionate treatment data for EE-TP to mediate metabolic correction of thymidine and deoxyuridine levels in patients with MNGIE. Based upon pre-clinical data and compassionate treatment data, the 24 month duration of the study is considered sufficient to assess the safety and tolerability, and to establish the pharmacodynamic effects of EE-TP. There is very limited natural history data available for MNGIE, and no clinical study data to determine the duration of treatment required to detect potential treatment effects. An interim review of open label data will be conducted after each of the 12 adult treatment naïve patients have been exposed to EE-TP for at least one year.

The rationale for the pharmacodynamic objective is based on the biochemical hallmark of MNGIE, where the deoxynucleosides thymidine and deoxyuridine accumulate in the plasma as a result of the deficient thymidine phosphorylase. The accumulation of thymidine impacts deoxynucleoside and nucleotide pools, and is thought to impede mtDNA replication and/or repair leading to mtDNA abnormalities and ultimately, mitochondrial dysfunction. Erythrocyte encapsulated TP is designed to replace thymidine phosphorylase in a sustained manner to reduce or eliminate elevated plasma thymidine and deoxyuridine concentrations. Pre-clinical studies have shown that thymidine and deoxyuridine diffuse across the erythrocyte membrane into the cell, where the encapsulated enzyme catalyses its metabolism to the normal products (thymine and uracil). This should result in a withdrawal of thymidine and deoxyuridine from the cellular compartments, and an amelioration of the intra mitochondrial deoxyribonucleotide imbalances. We hypothesise that by preventing further damage to mtDNA, mitochondrial turnover should allow mitochondrial regeneration, and thus the restoration of mitochondrial function [22]. As an exploratory objective, this study will also assess markers of mitochondrial condition, e.g., FGF21, GDF15, including mtDNA, mRNA, miRNA, and lipidomics/proteomics, and the potential effects of reducing thymidine and deoxyuridine levels on these markers. The pharmacodynamic and clinical data from the retrospective HSCT study supports the hypothesis that biochemical improvement results in clinical improvement, particularly in BMI, and supports the use of pharmacodynamic data as a biomarker for clinical efficacy [13].

Due to the absence of previous data on outcome measures in MNGIE, validated secondary outcome measures, including a quality of life measure, have been selected on the basis of clinical trials in similar disorders. We have chosen not to composite outcome measures due to the marked phenotypic variability of the disorder. No validated composite outcome measure exists for MNGIE. Using assessment/rating scales for mitochondrial disorders more generally (e.g., the Newcastle Mitochondrial Disease Assessment Scale) would be a risk in a very small population, with composite outcomes showing no effect overall, while a more critical individual measure (e.g., BMI, or mortality), would show evidence of benefit [23].

There are generally no accepted endpoints for clinical studies in patients with MNGIE. This ultra-rare disease usually presents with a combination of cachexia, gastrointestinal dysfunction, and neuromuscular dysfunction. Although weight loss is one of the key features of MNGIE and has a major impact on functional status, individual weight loss trajectories are not typically available in published case series. Weight loss is clearly associated with increased morbidity and mortality, and a decreased quality of life in other chronic diseases for which weight loss is a primary manifestation, including cancer [24], chronic obstructive pulmonary disease [25], chronic heart disease [26], chronic kidney disease [27], and acquired immunodeficiency syndrome [28]. Overall, current evidence shows that weight loss is a valid prognostic marker and is associated with decreased quality of life across different conditions. Additionally, at least in patients with cancer, weight loss and symptoms associated with anorexia cachexia have been shown to have a psychosocial effect, not only on patients, but also on the caregivers and families of those affected [29].

Weight is an objective measurement, which is readily obtained without causing additional burden to patients. Objectiveness is a critical requirement within the context of the planned open label design of this study. Serial measurements of body weight offer the simplest screen for nutritional adequacy and change in nutritional status. The weight and height of a patient are used to formulate the BMI (i.e., calculated by an individual’s body mass divided by the square of his/her height (kg/m^2^)). Since weight loss is essentially a universal feature of MNGIE, any weight gain is highly likely to represent a very sensitive clinical objective. Consequently, the primary clinical endpoint in this study will be mean absolute change from baseline in BMI.

Most published case series show a mean BMI of approximately 14 kg/m^2^ in patients with MNGIE, e.g., reference [4] describes 35 patients of which 16 were living at the time. The average height and weight for males were 170 cm and 40.3 kg, respectively, whilst for females were 159.1 cm and 35.5 kg. In the weight range reported for patients with MNGIE, an increase in BMI of approximately 0.2 represents an increase in body weight of approximately 0.5 kg. This increase is well above the measurement error for medical grade scales and is likely to be above intra individual variation for human weights in the low range. Therefore, an increase in BMI of 0.2 kg/m^2^ will empirically be used as a minimum threshold in counting patients with weight increase. Owing to the extreme rarity of the occurrence of MNGIE, a stabilisation of ±0.2 kg/m^2^ in BMI will be deemed to be clinically significant, rather than statistically significant, since it is expected to halt further clinical deterioration. Weight loss is clearly associated with increased morbidity and mortality in a variety of other chronic conditions. If adult patients with MNGIE were able to maintain or slightly increase their BMI with long-term therapy with EE-TP, this stabilisation would highly likely manifest itself in decreased overall morbidity and mortality, and hence overall clinical benefit in this population.

There is limited precedent for weight measurement following intervention for patients with MNGIE. In a retrospective international study of 24 patients with MNGIE treated with HSCT, 7 patients survived more than two years after HSCT, and all gained weight (median weight increase was 2.5 kg at a median follow-up of 53 months) [13]. This weight gain occurred in parallel with increase in TP activity to normal levels and normalisation of thymidine and deoxyuridine levels. Due to the retrospective international nature of the study, no consistent formal clinical outcome measures were documented. Subjective evaluation of clinical examination in two year survivors suggested improvements in muscle strength and sensation, although this was not quantified. A patient who received orthotopic liver transplantation for MNGIE was reported to have cachexia and body mass/BMI monitored, although no improvement was noted [30].

Treatments targeting anorexia and cachexia in other disorders, such as oxandrolone, dronabinol, megestrol acetate, growth hormone, and anamorelin, have previously used weight gain, with lean body mass and handgrip strength being used more recently. The mechanism of action of those products, targeting appetite and hormonal control of food intake, suggests that they would not be effective in patients with MNGIE.

Likewise, there is no systematic information on the illness experience of patients with MNGIE, and no disease specific instrument to collect it. The disease is a multi-system organ disorder with a heterogeneous presentation. Secondary outcome measures have been selected to assess gastrointestinal and peripheral sensorimotor polyneuropathy symptoms. The selected instruments are not disease specific, but have been shown to correlate significantly with both generic and disease targeted legacy instruments, and demonstrate evidence of reliability [31]. In addition, patient global impression of improvement, as well as improvement of the most disabling symptom for each patient, will be assessed.

The precise mechanism through which correction of the enzymatic defect through EE-TP therapy can decrease the elevated circulating levels of thymidine and deoxyuridine, ultimately restoring gastrointestinal function leading to an increase and stabilisation in body weight and improved outcomes, is speculative at this point. Previous research has shown that severe depletion of mtDNA is the main molecular defect in the gut wall of patients with MNGIE [21,32]. The depletion of mtDNA correlates with histopathological abnormalities and is likely due to toxic levels of thymidine and deoxyuridine disrupting the mitochondrial nucleotide pool through an unknown mechanism. The reduction of circulating levels of thymidine and deoxyuridine could, in principle, prevent further mtDNA depletion and, in time, promote the restoration of depleted gastrointestinal smooth muscle mtDNA pools. The consequent potential improvement of intestinal motility and improvement of nutrient absorption can be hypothesised to at least prevent further deterioration of the nutritional status and reduce gastrointestinal symptoms.

In summary, cachexia is the most consistent clinical manifestation of MNGIE. A robust clinical outcome measure of mean absolute change in BMI from baseline at 24 months is the primary endpoint. Stabilisation in BMI over a 24 month period will be regarded as a significant clinical benefit based on the consistent loss in BMI with time in patients with MNGIE. Relevant secondary endpoints included are the proportion of patients who require TPN, handgrip strength (using a dynamometer, according to the Southampton protocol), functional disability assessed by RODS, 10 m walk test, evaluation of gastrointestinal symptoms (PROMIS short form), neurological examination (for the assessment of presentation, improvement, or worsening in motor strength, distal sensory impairment, or areflexia), PGIC, assessment of the most disabling symptom for each patient using VAS, and quality of life. This range of secondary outcome measures will enable capture of clinical effect in a condition for which there is considerable phenotypic variability. 

## 4. Conclusions

To conclude, the open label, multiple dose design of this study will allow maximal data contributions from each patient in this ultra-rare indication, and offer sufficient flexibility to make use of continuously emerging knowledge that is generated as the study progresses. The design also incorporates safe progression of exposure, with multiple checks and provisions, ensuring risk is managed appropriately, i.e., all patients will receive the lowest dose of EE-TP for the first two doses with 24 h observation following exposure to the first four doses and Patient Oversight Committee recommendation regarding dose progression.

## 5. Patents

Bax, B.E and Bain, M.D. (2018) Treatment for mitochondrial neurogastrointestinal encephalomyopathy (MNGIE). EP2760459B1.

Bax, B.E and Bain, M.D. (2018) Treatment for mitochondrial neurogastrointestinal encephalomyopathy (MNGIE). US10213492B2.

## Figures and Tables

**Table 1:** Characteristics of EE-TP.

EE-TP Attributes	Criteria
Dosage Form	~58–65 × 10^10^ erythrocytes suspended in saline
Thymidine-phosphorylase/erythrocytes ratio	~30–90 U/10^10^ erythrocytes
Dosage Volume	~150–500 mL
Route of Administration	IV
Physical Description	Light to dark red color, resembling venous blood

EE-TP—erythrocyte encapsulated thymidine phosphorylase; IV—intravenous.

**Table 2:** Dose levels of EE-TP.

Dose Level	Erythrocyte Number and Thymidine Phosphorylase Activity (U *)
1 (low dose)	~58–65 × 10^10^ erythrocytes encapsulating 30–49 U TP/10^10^ erythrocytes
2 (mid dose)	~58–65 × 10^10^ erythrocytes encapsulating 50–69 U TP/10^10^ erythrocytes
3 (high dose)	~58–65 × 10^10^ erythrocytes encapsulating 70–90 U TP/10^10^ erythrocytes

* 1 unit of activity is defined as as the amount of enzyme required to convert 1 μmol of thymidine to thymine per min at 37 °C.

**Table 3:** Schedule of assessments from screening to the end of the run-in phase.

Assessment	Screening (Day −120 to Day −92)	Run-in Period (Day −91 to Day 0)		
		Day −91 to −84	Mid Run-in	Day −1 to 0
Informed consent	X			
Inclusion/exclusion criteria	X			
Demographics	X			
Medical history	X	X		
Anthropometrics ^1^	X	X	X	X
Viral serology	X			
Pregnancy test ^2^	X			
Serum follicle-stimulating hormone ^2^	X			
*TYMP* analysis	X			
Leucocyte TP activity	X			
Patient emergency card	X			
RBC count		X	X	X
Vital signs	X	X		
12-lead ECG	X	X		
Clinical laboratory evaluations ^3^	X	X	X	X
Haematocrit	X	X		
Physical examination	X	X		X
Plasma thymidine and deoxyuridine	X	X	X	X
Urine thymidine and deoxyuridine	X	X	X	X
BMI		X	X	X
Handgrip dynamometer		X	X	X
RODS		X	X	X
10-metre walk test		X	X	X
EuroQol-5D		X		X
PGIC		X		X
CGI-I		X		X
PROMIS		X	X	X
VAS ^4^	X	X		X
TPN use		X	X	X
Neurological examination		X		X
MRI (brain)		X		X
Abdominal ultrasound		X		X
Nerve conduction and electromyography ^5^		X		X
Neuro-ophthalmological assessment		X		X
FGF-21, GDF15, and other markers of mitochondrial condition		X		X

^1^ For adult patients, height will be measured at the beginning of run-in period. For juvenile patients, height will be measured with weight at all time points. ^2^ Female patients only; pregnancy test at Day 0 (Treatment Cycle 1) to be conducted prior to study drug administration. ^3^ Serum biochemistry: Alanine aminotransferase, aspartate aminotransferase, alkaline phosphatase, albumin, gamma glutamyl transferase, sodium, potassium, chloride, calcium, magnesium, inorganic phosphate, glucose (non-fasting), urea, uric acid, total bilirubin, direct bilirubin, creatine kinase, iron, ferritin, folate, vitamin b12, total protein, cholesterol (including total, low density lipoprotein, and high density lipoprotein), triglycerides, lactate dehydrogenase, c reactive protein, and creatine kinase. Haematology: white blood cell count, red blood cell count, red cell distribution width, haemoglobin, haematocrit, mean cell volume, mean cell haemoglobin, mean cell haemoglobin concentration, platelet count, white blood cell differential, international normalised ratio, CD4+, and CD8+. ^4^ At screening the investigator and patient will identify the patient’s most disabling symptom. ^5^ Sensory bilateral nerves: Median, ulnar, radial, sural, and superficial peroneal; motor bilateral nerves: Median, ulna, common peroneal, and post tibial; EMG: Dorsal interosseous, tibialis anterior, and gastrocnemius bilateral muscles.

**Table 4:** Schedule of assessments during the treatment and follow-up phases.

Assessment	Study Day																	
	0 TC 1	1	7	14	21 TC 2	22	28	35	42 TC 3	43	49	56	63 TC 4	64	70	77	Every 2–4 Weeks up to 24 Months	Follow-Up 90 Days Post-Dose
Pregnancy test ^1^	X						X					X					X	X
RBC count					X				X								X	
Administration of Study Drug	X				X				X			X					X	
Adverse event recording	Ongoing during the study																	
Concomitant medication ^2^	Ongoing during the study																	
Vital signs	X	X			X	X			X	X			X	X	X	X	X	X
12-lead ECG	X	X			X	X			X	X			X	X	X	X	X	X
Clinical laboratory evaluations ^3^	X		X		X	X									X	X	X	X
Physical examination					X										X	X	X	X
Anti-TP antibody/neutralising antibodies	X								X				X				X	X
Plasma thymidine and deoxyuridine			X	X	X		X	X	X		X	X	X		X	X	X ^4^	X
Urine thymidine and deoxyuridine	X		X	X	X		X	X	X		X	X	X		X	X	X ^5^	X
Body weight and height																	X	X
BMI																	X	X
Handgrip dynamometer													X				X	X
RODS													X				X	X
10-metre walk test													X				X	X
EuroQol-5D													X				X	X
PGIC													X				X	X
CGI-I													X				X	X
PROMIS													X				X	X
VAS symptom													X				X	X
TPN use													X				X	X
Neurological examination																	X	X
MRI (brain)																	X ^6^	X
Abdominal ultrasound																	X ^6^	X
Nerve conduction and electromyography ^7^																	X ^6^	X
Neuro-ophthalmological assessment																	X ^6^	X
FGF21, GDF15, and other markers of mitochondrial condition		X	X	X	X	X	X	X	X	X	X	X	X	X	X	X	X	X

^1^ Female patients only will be assessed at every 4 weeks. ^2^ Medication will be assessed for potential mitochondrial toxicity. ^3^ As reported in Table 4. ^4^ For the first 6 months, patients will return for an additional analysis of plasma thymidine and deoxyuridine at 7 days post-dose of every other treatment cycle. For rest of dosing, patients will return for an additional analysis of plasma thymidine and deoxyuridine at 7 days post-dose once every 4 treatment cycles. ^5^ 24 h urine collection for creatinine, thymidine, and deoxyuridine excretion will start from 6 days post-dosing for 24 h at every 4 treatment cycles. ^6^ Performed at 12 and 24 months. ^7^ As reported in Table 3. TC—treatment cycle.
